# The Electrical Aspect of Cataphoresis

**Published:** 1896-06

**Authors:** L. E. Custer

**Affiliations:** Dayton, Ohio


### THE DENTAL REGISTER.

VOL. L.] JUNE, 1896. [No. 6.

### COMMUNICATIONS.

### The Electrical Aspect of Cataphoresis.

BY L. E. CUSTER, B.S., D.D.S., DAYTON, OHIO.

Read before the Mississippi Valley Dental Society.

During the past year the literature and discussions upon cataphoresis have developed so many remarkable statements from an electrical point of view that it seems in place to give a few personal observations in this connection.

Since cataphoresis is a modification of the electrolytic property of electricity, the current must flow in one direction It may be continuous, pulsating or interrupted, but so long as it flows in one direction when it is in action, the result will be the same, and a suitable agent will be carried with it in cataphoresis. It has long been observed, however, that the more uniform the pressure is maintained on a continuous current the less it is felt in its various applications in electro-therapeutics and in proportion as the pressure varies, while it may still be continuous, will it be painful to the patient. So it may be stated that under steady pressure a small continuous current is not painful but becomes so when it pulsates. It becomes more so when it is interrupted and still more so when reversed in direction. It is for this reason that the interrupted current is used in shocking machines and that the alternating current is so deadly. Dr. Morton and others prefer the galvanic current partly for the steady voltage which is characteristic of this current and the small amount of pain accompanying its applications. On the other hand there are those who claim that the Edison current furnishes a practically steady voltage. Theoretically, the galvanic current gives the more uniform voltage, but those who use it seem to forget that they annull this virtue of battery power every time they touch the rheostat for increasing the current as is customary in its application.

The Edison current is ordinarily supplied at 110 volts pressure. In our city I have noticed by my volt meter that during the whole twenty-four hours it does not vary five volts and that during the daytime especially it does not vary two volts. The widest variations occur between 4:00 and 10:00 P. M. Now, when this current is used for cataphoresis by the appliance which I have devised for that purpose, by which I get the cataphoric current by a shunt circuit, the variation in voltage at the poles is in proportion, as the cataphoric voltage is to 110. For instance, if the Edison voltage is 110 and the cataphoric voltage average ten during the application, it would be necessary that the Edison current vary eleven volts to produce one volt variation in the shunt, or in other words, a variation of say five volts, an unusually large variation, in the main, would produce a variation of but half a volt at the cataphoric poles. Yet why object to this when the operator increases the voltage from one to five volts at each manipulation of the rheostat?

Altogether the objection to the Edison circuit, that its unequal voltage is perceptibly felt, does not appear as strong as claimed. It may be that where the poles of some appliances are directly in the circuit and in series with high resistance that the variation in the voltage is perceptibly felt, but in the appliance which I here present the variation averages very much less than one volt during an application and this is not perceptable. In many cases it is necessary to increase the pressure two to five volts before the patient feels the increase.

Many of the reports of cases have varied so much from the common electrical formula and from my own observations that either the measuring instruments were not correct or were not properly used. In one instance a writer speaks of increasing the current without increasing the voltage at the same time, while the resistance remained the same, notwithstanding the fact that electricians have for seventy years been substantiating the law laid down by Dr. Ohm, that the current strength in any circuit is equal to the electro-motive force divided by the resistance. It is not possible to increase the current through a constant resistance without increasing the pressure, or to use a common illustration, you can not increase the flow of water through a pipe of a given size without increasing the pressure.

The wide difference in the amount of current also led to some experiments as to their cause. The enamel consists of about 97 percent lime salts, which is a non-conductor of electricity, and the remaining 3 percent is such a small amount that it offers so much resistance as to be practically a non-conductor. This fact, that a sound tooth is covered by a non-conductor of electricity, may have a wider significance than we at first thought, would allow, and it may have a physiological significance as well. At any rate the fact that enamel is a non-conductor of electricity is an important consideration in cataphoresis.

On the other hand, about one-third of the dentine is made up of animal matter which contains water and is a comparatively good conductor. The matrix of the dentine is almost solid lime structure, and like enamel is a non-conductor. But within the tubule is contained the dentinal fibril which is made up almost entirely of water. It is this that we wish to obtund, and fortunately its large percentage of water makes it a good conductor. When the current is applied it follows the course of these canals to the pulp. Here I will call attention to the importance of enlarging the cavity with the chisel at first, about as it is desired when finished, because the fibrils anastomose so little that the area of anæsthesia is confined almost entirely to those tubuli whose mouths open into the cavity. In a long application in a deep cavity, however, the pulp becomes affected and the fibrils supplied to other portions of the crown lose their sensation as deeply down as the pulp itself has been affected.

So then the enamel being a non-conductor of electricity and the path of the current following the tubuli through the dentine, it is easy to understand why in some instances an operator could use one milliampere while in another but one-tenth as much as the same voltage. A small exposure of dentine is like a small wire, it offers more resistance than a large cavity or a large wire. In the practical operation of cataphoresis we must consider the path from the positive pole in the cavity to the negative pole at the sponge to be like a funnel with the small end equal to the area of exposed dentine and the large end the area of the sponge upon the face. No more water can flow through the funnel than can pass through the smaller end, and so no more current can flow than can pass through the exposed tubuli, and the current increases in proportion to the size of the cavity at the same voltage. It is for this reason that an ammeter is not of much practical value for dentine anæsthesia. It is often a satisfaction, however, to know how much current is being used in the operation, but if it is to be used for recording and the establishing of tables the size of the cavity must also be tabulated.

Dentine which has been denuded of enamel for a long time will probably offer greater resistance than freshly-exposed dentine.

The position of the negative electrode has much to do with the application of the current. The shorter the distance this is placed from the tooth under operation the less voltage will be required to force the current through, and the less will be the variations due to the alleged unequal Edison current. After experiment I find that it requires about three times the voltage when the sponge is held in the hand than when held upon the cheek, so would recommend that the negative pole be placed upon the cheek and held in place by the same appliance which ordinarily holds the rubber dam back. This has been my method, and it is seldom that the voltage exceeds fifteen, while in some accounts I see it is very much more than that.

As to the detail of manipulation it is essential that the current be not broken during the application. This is very likely to occur if the anode is held by the operator. Even if the instrument does not come entirely out of the cavity it is very difficult to hold it perfectly quiet for the ten or fifteen minutes of the application. The least movement is perceptible to the patient, and especially if the cotton is not well saturated. To avoid any such dangers I have devised an anode to be slipped on the ordinary rubber-dam clamp. This appliance is so constructed that the anode is electrically insulated from the clamp and at the same time is firmly held in position. This allows the operator free to manage the administration of the current. In this connection I might state that appreciating the fact that a gradually increased current is much less painful than one by steps. I hope soon to complete an appliance actuated by clock-work which will meet the requirements of dental cataphoresis.

It may be worth while to call attention to the placing of the anode in the cavity. It is to be inferred from reports that the operator generally places a saturated pellet of cotton in the cavity and then holds the anode upon that. The reverse should be the order. Secure the instrument in the cavity and place a heavily saturated pellet of cotton about it and the instrument will be flooded about by capillary attraction, and any small movement of the instrument will not be perceptible, whereas if it is placed upon the cotton the loss by electrolysis is noticeable and there is greater danger of breaking the current.

The appliance which I have devised for this purpose is made in this manner: one 8 c. p., or preferably two 16 c. p., lamps are used for the main resistance. Two 16 c. p. lamps are preferred only for safety, as a ground would meet with resistance on either wire. In series and between the two lamps is placed a resistance coil of about sixty feet of .01 German silver wire wound in a No. 34 thread cut in a rod of fiber an inch in diameter and seven inches in length. To the wire to the left is attached the wire leading to the cathode. An ammeter may be placed anywhere in this wire. The wire from the anode is attached to a movable brush which slides along in contact with the resistance coil. If the anode is placed in the cavity and the cathode upon the cheek a circuit from the brush will be closed through the patient as well as the one through the main line. The circuit through the patient is called a shunt circuit. When the brush is at the left, no current flows through the patient because the patient offers so much resistance and there is no resistance on the main wire, but on moving the resistance between the two points increases so much that the current divides and a little is sent through the patient. The amount of current flowing through one branch of a divided circuit is inversely proportionate to the resistance of the other branch, so that if the resistance increase in one branch more will be forced through the other. So by this arrangement if 16 c. p. lamps are used, as illustrated, there will be a range of twenty-four volts through the patient. If instead of 16 c. p. lamps, 20 c. p. lamps are used, there will be a range of about thirty volts, and so on.

Such an appliance can be made at a very small cost, and since the operator is guided by signs from the patient how much current to use, the volt meter and ammeter are not strictly necessary.

DISCUSSION.

[The Executive Committee had made arrangements with a number of patients to be present in the operating room of the college as subjects for illustrating Dr. Custer's method of cataphoresis. Upon the conclusion of the paper the association adjourned in a body to the commodious Infirmary and witnessed the clinical demonstration.]—REP.

DR. H. A. SMITH: Dr. Custer's paper is so complete that discussion of it seems to be superfluous. In connection with this subject it occurs to me that a 25 percent solution or mixture of pyrozone in a dead tooth would, by applying the current, liberate nascent oxygen and an instantaneous bleaching would result. I can not see why complete disinfection of root-canals might not be accomplished in the same way.

DR. J. S. CASSIDY: This paper is above criticism. In the process known as electrolysis, the electrolyte is decomposed. What I would like to satisfy my mind upon is, will cataphoric action carry in the medicament without decomposing it? Will electrolysis interfere with cataphoresis? Is the cocaine decomposed? If, in using hydric-peroxide, would the oxygen be carried through the tooth if the poles were reversed, oxygen always going to the positive pole?

DR. O. N. HEISE: The subject, as presented by Dr. Custer, is mainly in its relation to the kind of apparatus used. He does away with the expensive ones now on the market like the Wheeler Fractional Volt Selector, which, no doubt, has been the one mostly used and mentioned in the various articles published on this subject; the cost of this apparatus is sixty-five dollars. The one here spoken of is simplicity itself, and every one who so desires can make one for himself and at an expense of a few dollars. I hope, however, that Dr. Custer will take the trouble to put them on the market.

One of the points in the paper, and a very important one in the practical and successful application of cataphoresis, is his calling attention to the non-conductivity of the enamel. If we should attempt to obtund the dentine by placing the positive pole on the enamel, and proceed in the usual manner, our success would be *nil* and only lead us to lose confidence in cataphoresis. I have heard of some parties making the bold statement that they could force the cocaine solution through the enamel without any difficulty. I think it was a lack of experience that led them to make such a statement.

The other point, as to the size of cavity and number of dentinal tubuli exposed, has a direct bearing on the success of its application. The dentine itself is a poor conductor of electricity, and the more we can open a cavity by cutting away the enamel, that much more readily and positively can we force the cocaine solution through the tubuli and thereby have its obtunding effects produced. The subject of cataphoresis and electrolysis is one of vast importance to us, and I hope that Dr. Custer, who has done so much in this intricate subject of electricity, will go on with his experimentations, and I know his results will be fruitful and beneficial to the profession.

Dr. Custer: Some one has asked the question as to application for extraction. For this purpose two branches of the positive pole are to be applied one on each side opposite the root apex, the negative on some other convenient part of the body.

Dr. E. G. Betty: Dr. Custer, will cataphoric action take place when the positive pole is applied to an unbroken surface of enamel?

Dr. Custer: I have found that I was unable to drive a 40-volt current through enamel, though the tooth had not been dried, the rubber dam having just been adjusted. Anæsthetization can not take place through enamel, it being so poor a conductor as not to be a conductor at all.

Subject passed.

